# Retroperitoneal Fibrosis: A Rare Cause of Acute Renal Failure

**DOI:** 10.1155/2012/645407

**Published:** 2012-07-15

**Authors:** Amaka Ezimora, Marquetta L. Faulkner, Oluwafisayo Adebiyi, Abimbola Ogungbemile, Salas-Vega Marianna, Chike Nzerue

**Affiliations:** Department of Internal Medicine, Meharry Medical College, Nashville, TN 37208, USA

## Abstract

*Introduction*. Retroperitoneal fibrosis is a rare cause of acute renal failure (ARF) with only a handful of cases reported in literature. We report a case of a 40-year-old male with an incidental finding of retroperitoneal fibrosis. *Case Presentation*. Patient is a 40-year-old African American male with no significant past medical history who presented with a four-month history of low back pain and associated nausea with vomiting. Physical examination was significant for elevated blood pressure at 169/107 mmhg and bilateral pedal edema. Significant admission laboratory include blood urea nitrogen (BUN) of 108 mg/dL, serum creatinine (Cr) of 23 mg/dL, bicarbonate of 19 mg/dL, and potassium of 6.2 mmL/L. Renal ultrasound showed bilateral hydronephrosis. Post-void residual urine volume was normal. Abdominopelvic CT scan showed retroperitoneal fibrosis confirmed with fine-needle biopsy. He was treated with a combination of bilateral ureteral stent placement, hemodialysis, and steroid therapy. Four months after hospital discharge, his BUN and Cr levels Improved to 18 mg/dL and 1.25 mg/dL, respectively. *Conclusion*. Retroperitoneal fibrosis should be considered as a differential diagnosis in patients with acute renal failure and obstructive uropathy. Abdominal CT scan is the examination of choice for diagnosis. Full resolution with treatment depends on the duration of obstruction.

## 1. Introduction/Epidemiology

Retroperitoneal fibrosis (RF) is a rare disorder with one Finnish study showing a prevalence of 1.4/100,000 inhabitants and an incidence of 0.1/100,000 person-years [1]. However, in another study, a higher incidence of 1.3/100,000 was reported [2]. It is characterized by the presence of a retroperitoneal tissue, consisting of chronic inflammation and marked fibrosis, which often entraps the ureters or other abdominal organs [3]. Two forms of the disorder have been described in published literature. The idiopathic form of the disease accounts for more than two thirds of cases, with the rest being secondary to other factors—for example, neoplasm, infections, trauma, radiotherapy, surgery, and use of certain drugs [4].

Idiopathic disease most commonly occurs in individuals 40 to 60 years of age, with a 2 to 3 : 1 male-to-female predominance [4]. The first description of idiopathic retroperitoneal fibrosis is credited to the French urologist Albarran [4] who, in 1905, reported the surgical treatment of an extensive fibrotic retroperitoneal process causing ureteral obstruction [5, 6]. Reports describing idiopathic retroperitoneal fibrosis first appeared in the English medical literature in 1948 [7]. This disorder has also been referred to as Ormond's disease, periureteritis fibrosa, periureteritis plastica, chronic periureteritis, sclerosing retroperitoneal granuloma, and fibrous retroperitonitis.

Renal failure is a common finding in a patient with idiopathic retroperitoneal fibrosis (IRF). About 80–100% of IRF patients show ureteral involvement: this is bilateral in most cases, and in patients with unilateral obstruction contralateral disease may develop within weeks to months. Additionally, some patients present with nonfunctioning kidneys, as a result of long-lasting obstructive uropathy [8–11].

## 2. Case Report

A 40-year-old African American male with no significant past medical history presented with 4-month history of low back pain and associated nausea with vomiting. He admitted to poor urinary stream but no oliguria, dysuria, or hematuria.

Physical examination at presentation was significant for elevated blood pressure of 169/107 mmHg. No back or abdominal tenderness, organ enlargement, or palpable mass was elicited. Trace pedal edema was noted bilaterally.

**Figure 1:**
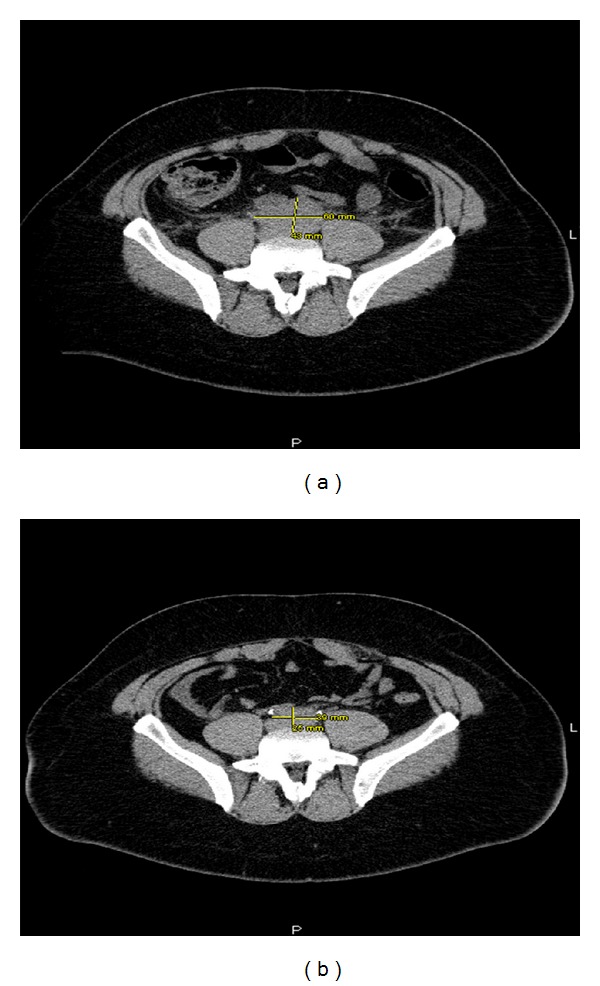
Abdominal CT scan showing retroperitoneal fibrosis at diagnosis and four months after operation.

Laboratory results showed blood urea nitrogen (BUN) of 108 mg/dL, serum creatinine (Cr) of 23 mg/dL, bicarbonate of 20 mg/dL, and potassium of 6.2 mmL/L. Renal ultrasound showed moderate bilateral hydronephrosis with no identifiable cause. Hemodialysis was started because of his worsening renal function. After dialysis, there was a mild improvement of his BUN/Cr with resolution of his hyperkalemia and symptoms. However, the patient was noted to be oliguric. Post-void residual urine volume was normal. CT of the abdomen and pelvis was obtained for further evaluation of the obstruction which showed a retroperitoneal lobulated mass measuring 4 cm × 6 cm, producing obstruction to the lower aspects of the bilateral ureters (Figure 1(a)).

**Figure 2:**
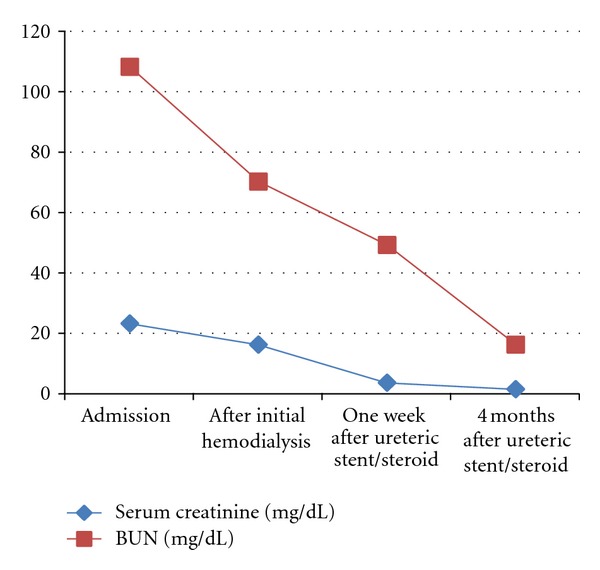
Patient's BUN and serum Cr levels trend.

Confirmatory diagnosis was made with a CT-guided fine needle biopsy which showed collagen-rich fibroblastic elements with diffuse inflammatory infiltrates. The cytology was negative for malignancy. He was treated with bilateral ureteral stent placement to relieve the obstruction in addition to hemodialysis and intravenous methylprednisolone. There was a significant improvement of his renal function evidenced by his decreasing BUN and Cr levels (Figure 2) and good urinary output. Thereafter, hemodialysis was discontinued and the patient was maintained on oral prednisone. Subsequent laboratories included antineutrophilic antibodies, proteinase 3, and hepatitis C antibodies, which were negative.

He was followed up with serial abdominal CT scan. At four months after operation, the abdominal CT scan showed near resolution of his bilateral hydronephrosis and marked reduction in the size of the retroperitoneal mass (Figure 1(b)). In addition, his BUN and serum Cr levels improved to 18 mg/dL and 1.25 mg/dL, respectively.

## 3. Pathogenesis

The pathogenesis of the idiopathic disease is unclear. The two leading theories are

- an exaggerated local inflammatory reaction to aortic atherosclerosis, incited by oxidized low density lipoprotein (LDL)—this mechanism is supported by the finding in patients with chronic periaortitis (whose spectrum includes IRF) of activated T and B lymphocytes in the media and adventitia, high levels of interleukins in aortic wall sections, and circulating antibodies to oxidized LDL [8, 9].
- A manifestation of systemic autoimmune disease—this hypothesis is supported by the frequent presence of constitutional symptoms, raised concentrations of acute phase reactants, autoantibodies, and known autoimmune disease.

The most common causes of secondary retroperitoneal fibrosis (Table 1) are medications like ergot derivatives, beta blockers, hydralazine, and analgesic. Ergot derivatives like methysergide can cause fibrotic reactions that affect not only the retroperitoneum, but also the pericardium, the pleura, and the lungs. Other causes of secondary RF include malignancies, infections, radiotherapy, and surgery [7]. Serotonin mediated mechanism has been suggested whenever ergot derivative and carcinoid are the identified secondary causes [11, 12].

## 4. Clinical Presentation

Retroperitoneal fibrosis often presents with nonspecific clinical signs and symptoms which are secondary to both the local and systemic effects. The most common localized symptoms are abdominal and back pain [3, 6, 11]. Extrinsic compression of retroperitoneal lower limb lymphatics and venous system manifest as lower limb edema, scrotal edema, varicocele, and deep venous thrombosis. Other symptoms reported include constipation, small bowel obstruction, hematuria, and oliguria whenever there is bilateral ureteral obstruction [13].

The systemic manifestations are often nonspecific and include constitutional symptoms such as fatigue, low-grade fever, nausea, anorexia, weight loss, and myalgia [9]. Some patients with bilateral ureteral obstruction can present with uremic symptoms. Physical examination may reveal abdominal or back tenderness or abdominal mass. Abdominal bruit may also be heard if the aorta is involved in the entrapment [6].

## 5. Autoimmune Diseases Associated**** with Retroperitoneal Fibrosis

Patients with idiopathic retroperitoneal fibrosis often have mild manifestations of an autoimmune disease. However, the frequency of the association between retroperitoneal fibrosis and other autoimmune diseases is unknown. The most important associated autoimmune diseases are listed in Table 2.

## 6. Diagnosis

The laboratory findings often reflect an acute-phase reaction, with high erythrocyte sedimentation rate (ESR) and C-reactive protein (CRP) levels [9]. Autoantibodies like antinuclear antibodies are also sometimes positive in patients with idiopathic type. Azotemia may be present depending on the extent of ureteral obstruction. Additionally, some patients present with nonfunctioning kidneys, as a result of long-lasting obstructive uropathy [3, 9].

Image evaluation is accomplished with sonography in patient presenting with azotemia. Abdominal ultrasound reveals retroperitoneal fibrosis as hypoechoic or isoechoic mass, which can involve the ureters and, thus, cause unilateral or bilateral hydronephrosis [6]. CT and MRI are the most reliable modality for diagnosis because they are noninvasive and provide a picture of the disease, which is typically that of a periaortic mass of soft-tissue density extending from the level of the renal arteries to the iliac vessels and frequently causing medial ureteral deviation and obstruction. Atypical peripancreatic, periureteral, or pelvic localizations have also been reported [11, 14]. Ureteral obstruction causing acute or chronic renal insufficiency is the most common and severe complication of IRF.

About 80–100% of idiopathic patients show ureteral involvement: this is bilateral in most cases, and in patients with unilateral obstruction contralateral disease may develop within weeks to months [8].

Histology is often needed to confirm diagnosis and to rule out other differentials. A collagen-rich background with fibroblastic/myofibroblastic elements lacking signs of atypia, mitotic figures, or morphological or immunohistochemical features of other fibrous proliferations, along with a diffuse and perivascular inflammatory infiltrate mainly consisting of T and B lymphocytes (but also including macrophages, IgG4-positive plasma cells, and eosinophils) may be considered the histopathological portrait of IRF [8].

There are no guidelines concerning the need for a biopsy to confirm diagnosis except if the location of the mass is atypical, clinical, laboratory, or radiologic findings suggest the presence of an underlying malignancy or infection, limited local experience in the diagnosis of retroperitoneal fibrosis and in situations where patient does not respond to initial therapy [6].

## 7. Treatment

Treatment of RF depends on whether it is idiopathic or secondary. Treatment modalities available include medical and surgical therapies. The goals of therapy are to relieve the obstruction, stop the progression of the fibrotic process, and prevent disease relapse. Glucocorticoids are considered the mainstay of therapy for IRF. In secondary causes, treatment is aimed at the underlying etiology or stopping the offending drug. Additional treatment with steroid may be beneficial in drug-induced RF if there are severe disease manifestations or no significant clinical improvements despite discontinuing the drug [13]. However, there are no established predictors of response to glucocorticoid therapy. Furthermore, the optimum dose and duration of steroid therapy are not well established because it has not been tested in a controlled trial.

An initial prednisone dose of 1 mg/kg per day (maximum dose 80 mg/day) is usually administered for approximately 4–6 weeks. Thereafter, tapering dose can be maintained for about 1-2 years depending on the disease progress [6, 15, 16]. If the disease does not adequately respond to steroid therapy alone, immunosuppressive agents can be used concurrently with steroid. Agents that have been used with apparent success in case reports and case series include Azathioprine, Methotrexate, mycophenolate mofetil, cyclophosphamide, and cyclosporine [10, 17–19]. However, no randomized studies have compared the effectiveness or toxicity of various immunosuppressants plus steroids versus steroids alone. In addition, some cases [20–22] have reported positive responses to tamoxifen alone as first line therapy but its actual effectiveness is uncertain because of lack of larger studies.

Surgical intervention is indicated in patients presenting with renal failure due to urinary tract obstruction or with unilateral severe kidney obstruction even if renal function is normal. In addition, lack of regression of the retroperitoneal mass after medical therapy will require surgical treatment. However, surgery does not prevent disease progression or recurrence and it has no effect on systemic manifestations. Conservative approach with temporary placement of ureteral stents or nephrostomy tubes is recommended for definite cases of idiopathic retroperitoneal fibrosis with obstruction [6, 16]. Open surgical approach: ureterolysis with intraperitoneal transposition and wrapping of the ureters with omental fat is reserved for patients with technical difficulties or in whom investigative and clinical findings are suggestive of underlying malignancy, in order to obtain a definite diagnosis [3, 11, 23]. Laparoscopic ureterolysis has been reported as a feasible approach and may be associated with a shorter hospital stay and reduced need for blood transfusion [23, 24]. However, certain risks and complications including ureteric leakage, thromboembolism, and recurrent fibrosis have been associated with these procedures [17]. Thus, patients with mild unilateral hydronephrosis without significant renal impairment can be closely monitored on medical therapy alone and later referred for surgical decompression if renal function deteriorates or hydronephrosis worsens.

## 8. Followup

After the initiation of medical therapy, patients are monitored clinically for resolution of symptoms with regular assessment of the erythrocyte sedimentation rate, C-reactive protein and serum creatinine concentration. In addition, serial CT scan is used to monitor the size of the fibrotic mass. Ultrasonography is useful in the followup of ureteral obstruction after the surgical decompression. It is also used to evaluate for obstruction, especially if a procedure was deferred or following removal of nephrostomy tubes or stents. With effective treatment, full resolution of the mass may occur. On the other hand, treatment failure after 4–6 weeks of therapy should prompt reevaluation with CT scan and biopsy for definite diagnosis. In addition, an increase in mass size or a new complication related to the mass may prompt surgical intervention. Thus, indefinite surveillance is recommended since disease relapse has been reported up to 10 years after discontinuation of therapy. In cases where residual tissue remains, positron emission tomography with 18F-fluorodeoxyglucose (FDG) is useful to assess the metabolic activity of the tissue [25]. Very little or absent FDG accumulation is suggestive of an inactive residual disease.

## 9. Prognosis

Prognosis in most patients is considered to be good [3]. However, no predictors of response to therapy, corticosteroid requirement, or disease relapse have been identified [6]. The relapse rate after discontinuation of treatment ranges from less than 10–30%, although one series reported a recurrence rate of over 70% [10, 26–29]. Ureteral obstruction is estimated to recur in up to half of patients who undergo surgery alone and in about 10% of those treated with both surgery and steroid [10]. Permanent renal damage leading to chronic renal failure requiring dialysis may occur. In glucocorticoid-responsive diseases excluding cases associated with malignancy, mortality rate is 10% over many years of followup [15].

## Figures and Tables

**Table 1:** Major causes of secondary retroperitoneal fibrosis Adapted from [6].

Examples	
Drugs	Methysergide, pergolide, bromocriptine, ergotamine, methyldopa, hydralazine, analgesics, beta blockers
Malignant diseases	Carcinoid, Hodgkin's and non-Hodgkin's lymphomas, sarcomas, carcinomas of the colon, prostate, breast, stomach
Infections	Tuberculosis, histoplasmosis, actinomycosis
Others	Histiocytoses, Erdheim-Chester disease, amyloidosis, trauma, barium enema

**Table 2:** Main association between retroperitoneal fibrosis and autoimmune or inflammatory diseases. Adapted from [6].

Autoimmune thyroid disease
Hashimoto thyroiditis
Reidel's thyroiditis
Graves's disease

Small and medium-sized vessel vasculitis
Wegener's granulomatosis
Polyarteritis nodosa
Microscopic polyangiitis
Hepatitis c virus-related cryoglobulinemia

Glomerulonephritis
ANCA-positive rapidly progressive glomerulonephritis
Membranous nephropathy

Ankylosing spondylitis
Systemic lupus erythematosus
Rheumatoid arthritis
Sclerosing cholangitis
Uveitis
